# Population pharmacokinetics of oseltamivir and oseltamivir carboxylate in obese and non‐obese volunteers

**DOI:** 10.1111/bcp.12892

**Published:** 2016-03-04

**Authors:** Kalayanee Chairat, Podjanee Jittamala, Warunee Hanpithakpong, Nicholas P. J. Day, Nicholas J. White, Sasithon Pukrittayakamee, Joel Tarning

**Affiliations:** ^1^ Mahidol–Oxford Tropical Medicine Research Unit, Faculty of Tropical MedicineMahidol UniversityBangkokThailand; ^2^ Faculty of Tropical MedicineMahidol UniversityBangkokThailand; ^3^ Centre for Tropical Medicine and Global Health, Nuffield Department of MedicineUniversity of OxfordOxfordUK

**Keywords:** influenza, obesity, oseltamivir, population pharmacokinetics

## Abstract

**Aims:**

The aims of the present study were to compare the pharmacokinetics of oseltamivir and its active antiviral metabolite oseltamivir carboxylate in obese and non‐obese individuals and to determine the effect of obesity on the pharmacokinetic properties of oseltamivir and oseltamivir carboxylate.

**Methods:**

The population pharmacokinetic properties of oseltamivir and oseltamivir carboxylate were evaluated in 12 obese [body mass index (BMI) ≥30 kg m^−2^) and 12 non‐obese (BMI <30 kg m^−2^) Thai adult volunteers receiving a standard dose of 75 mg and a double dose of 150 mg in a randomized sequence. Concentration–time data were collected and analysed using nonlinear mixed‐effects modelling.

**Results:**

The pharmacokinetics of oseltamivir and oseltamivir carboxylate were described simultaneously by first‐order absorption, with a one‐compartment disposition model for oseltamivir, followed by a metabolism compartment and a one‐compartment disposition model for oseltamivir carboxylate. Creatinine clearance was a significant predictor of oseltamivir carboxylate clearance {3.84% increase for each 10 ml min^−1^ increase in creatinine clearance [95% confidence interval (CI) 0.178%, 8.02%]}. Obese individuals had an approximately 25% (95% CI 24%, 28%) higher oseltamivir clearance, 20% higher oseltamivir volume of distribution (95% CI 19%, 23%) and 10% higher oseltamivir carboxylate clearance (95% CI 9%, 11%) compared with non‐obese individuals. However, these altered pharmacokinetic properties were small and did not change the overall exposure to oseltamivir carboxylate.

**Conclusions:**

The results confirmed that a dose adjustment for oseltamivir in obese individuals is not necessary on the basis of its pharmacokinetics.

## What is Already Known about this Subject

- An increased risk of poor outcome has been observed in obese patients infected with A(H1N1) pdm09 influenza.
- Obese patients have a lower overall exposure to oseltamivir but a similar exposure to the active metabolite, oseltamivir carboxylate.
- The effect of obesity has been characterized previously in severely obese volunteers but not in a study in obese and non‐obese control subjects.

## What this Study Adds

- This was the first population pharmacokinetic study of oseltamivir in obese and non‐obese control subjects.
- Obese individuals had an approximately 25% higher oseltamivir clearance, 20% higher oseltamivir volume of distribution and 10% higher oseltamivir carboxylate clearance compared with non‐obese individuals.
- Creatinine clearance was a significant predictor of oseltamivir carboxylate clearance.
- This study confirmed that a dose adjustment for oseltamivir in obese individuals is not necessary.

## Introduction

A novel influenza virus, A(H1N1)pdm09, apparently of swine origin, emerged in March 2009 in North America and spread rapidly among humans around the world. The World Health Organization (WHO) declared the end of the pandemic in August 2010, although A(H1N1)pdm09 continues to cause illness as a seasonal influenza virus. Poor clinical outcomes were observed in several patient populations infected with this virus – mainly geriatric patients aged ≥65 years, pregnant patients in the third trimester, obese patients and patients with concomitant chronic illnesses 1.

Oseltamivir is the most widely used antiviral agent for the treatment and prophylaxis of seasonal influenza and for A(H1N1)pdm09 influenza. The recommended oral dose for adolescents (13–17 years of age) and adults is 75 mg oseltamivir twice daily for 5 days, regardless of body weight or body mass index (BMI). Higher doses of oseltamivir and longer durations of treatment were recommended for the treatment of A(H1N1)pdm09 influenza‐infected patients with severe or progressive clinical presentations 2. However, previous studies suggested that a higher dose (i.e. 150 mg oseltamivir) provided no additional clinical benefit over the standard dose in patients with severe influenza 3, 4, 5.

Oseltamivir displays dose‐linear pharmacokinetics and is rapidly and almost completely absorbed 6, 7. It is bioactivated by hepatic carboxylesterase 1 (CES1) into its active metabolite, oseltamivir carboxylate, which is subsequently excreted by the kidney with a half‐life of 8 h 8. A longer terminal half‐life, of 6–10 h, for oseltamivir carboxylate after oral administration of oseltamivir, in comparison with 1.8 h following intravenous administration of the metabolite, suggests flip‐flop pharmacokinetics – i.e. parent‐to‐metabolite conversion being the rate‐limiting step of the elimination 7. Evaluating the proportion of oseltamivir and oseltamivir carboxylate excreted in the urine after oral administration showed that approximately 93% of the orally administered dose of oseltamivir is converted into oseltamivir carboxylate in healthy volunteers 9.

Data from many countries around the world indicated an association between obesity (BMI ≥30 kg m^−2^) and severe morbidity and increased mortality in patients infected with A(H1N1)pdm09 influenza virus 10, 11, 12, 13, 14. A cohort study over 12 influenza seasons (1996–1997 to 2007–2008) in Canada suggested that severe obesity (BMI ≥35 kg m^−2^) was associated with an increased risk for respiratory hospitalizations during influenza seasons 15. The mechanisms underlying this association have not been fully established. Obesity is associated with increases in both lean and fat masses as well as numerous physiological changes that can alter drug tissue distribution, hepatic metabolism and/or renal excretion, depending on the physical and chemical properties of the drugs 16, 17.

Previous studies have shown a significantly higher clearance of the inactive prodrug oseltamivir which subsequently led to lower oseltamivir exposure in healthy obese individuals. In addition, a modest decrease in oseltamivir carboxylate exposure in obese individuals was observed but was considered to be clinically insignificant. The study concluded that a dose adjustment in obese patients was not warranted 18, 19. A population pharmacokinetic model developed for healthy obese volunteers with a BMI of ≥40 kg m^−2^ suggested comparable pharmacokinetics to those from non‐obese individuals in another study 20. These authors showed that body size was significantly associated with the apparent clearance of oseltamivir and oseltamivir carboxylate but the lack of a non‐obese control group restricted the interpretation of these findings.

Non‐compartmental analysis comparing the pharmacokinetics of oseltamivir and oseltamivir carboxylate in obese and non‐obese Thai volunteers after a single oral dose of 75 mg and 150 mg of oseltamivir have been presented in full elsewhere 18. The aims of the present study were to characterize and quantify the effect of obesity on the pharmacokinetic properties of oseltamivir and oseltamivir carboxylate using nonlinear mixed‐effects modelling. The relationship between various size descriptors and pharmacokinetic parameters was assessed and quantified to enable a mechanistic understanding of the pharmacokinetic differences.

## Methods

### Study design and study participants

The study was an open‐label, crossover, randomized pharmacokinetic study conducted in 12 obese (BMI ≥30 kg m^−2^) and 12 non‐obese (BMI <30 kg m^−2^) healthy adult volunteers at a single study centre (Hospital for Tropical Diseases, Faculty of Tropical Medicine, Mahidol University, Bangkok, Thailand). Volunteers were healthy males and nonpregnant females aged between 18 and 60 years of age. Volunteers eligible to enter the protocol received a single oral dose of 75 mg and 150 mg of oseltamivir (fasted), in a random sequence, for two consecutive visits, with an intervening washout period of 7 days. Complete clinical details and noncompartmental analysis results are reported in full elsewhere 18. Protocol and amendments were reviewed and approved by the Faculty of Tropical Medicine Ethics Committee, Mahidol University and the Oxford Tropical Research Ethics Committee. The trial was registered at ClinicalTrials.gov (Clinicaltrials.gov identifier: NCT01049763).

### Drug analysis

Whole blood samples were drawn via an intravenous cannula. Blood (2 ml) was collected in fluoride–oxalate collection tubes at predose, and then 0.5, 1, 1.5, 2, 3, 4, 5, 6, 7, 8, 10, 12 and 24 h post‐dose. Samples were centrifuged for 7 min at 2000×*g* at 4°C. After centrifugation, plasma was transferred to prelabelled storage tubes and frozen in the upright position at −70°C until analysis.

Plasma samples were analysed for both oseltamivir and oseltamivir carboxylate using a validated assay employing automated solid‐phase extraction in the 96‐well plate format prior to separation with zwitterionic–hydrophilic interaction liquid chromatography coupled to tandem mass spectrometry 21. Quality control samples at low, middle and high concentrations for oseltamivir and oseltamivir carboxylate were analysed in three replicates within each analytical batch for method accuracy and precision. The coefficients of variation for oseltamivir analysis were 2.76%, 2.85% and 2.52% at 3 ng ml^−1^, 20 ng ml^−1^ and 150 ng ml^−1^, respectively. The coefficients of variation for oseltamivir carboxylate analysis were 2.42%, 2.50% and 1.96% at 30 ng ml^−1^, 400 ng ml^−1^ and 4000 ng ml^−1^, respectively. The lower limits of quantification (LLOQ) were 1 ng ml^−1^ for oseltamivir and 10 ng ml^−1^ for oseltamivir carboxylate.

### Pharmacokinetic analysis

Concentrations of oseltamivir and oseltamivir carboxylate were converted to equivalent molar units and transformed to natural logarithms before analysis. Concentration–time profiles of oseltamivir and oseltamivir carboxylate were modelled simultaneously with nonlinear mixed‐effects modelling using NONMEM version 7.2 (ICON Development Solutions, Ellicott City, MD, USA). Automation and data postprocessing was performed using Perl‐speaks‐NONMEM (PsN) version 3.6.2 and Xpose version 4.0 in the programming language R. Beal's M3 method was evaluated for handling concentrations below the LLOQ, which were treated as censored data 22, 23. The first‐order conditional estimation (FOCE) method with interactions or the Laplacian estimation methods for the M3 methodology were used throughout the study. Objective function value (OFV; proportional to twice the negative log‐likelihood of the data) was used to discriminate between two competing hierarchical models. A decrease in OFV of 3.84, 6.63 and 10.83 was considered a significant improvement at *P* < 0.05, *P* < 0.01 and *P* < 0.001, respectively, with one degree of freedom (one additional parameter).

The renal clearance of oseltamivir is negligible (i.e. 7%) and therefore was not included as a fixed assumption in the model 9. As oseltamivir carboxylate is the main product of oseltamivir metabolism, it was assumed that oseltamivir is metabolized completely into oseltamivir carboxylate. The structural base models were parameterized as first‐order absorption rate constant (k_a_), oral oseltamivir clearance (CL/F_OS_), oseltamivir volume of distribution (V/F_OS_), oseltamivir carboxylate clearance (CL/F_OC_), oseltamivir carboxylate volume of distribution (V/F_OC_) and relative oral bioavailability (F).

Interindividual random variability was modelled exponentially for all parameters, as shown in equation 1:

(1) $$\theta_{i\;}=\theta \times \exp\left(\eta_{i,\theta }\right)$$

where *θ*_*i*_ is the individual parameter for the *i*th subject, *θ* is the typical population value of the parameter and *η*_*i*,*θ*_ is the interindividual random variability with mean zero and variance Ω^2^. The residual random variability was modelled as separate additive errors on log‐transformed concentrations of oseltamivir and oseltamivir carboxylate, which essentially correspond to exponential residual errors on the normal scale.

One‐, two‐ and three‐compartment disposition models were evaluated. A transit compartment was added to accommodate a slower formation rate of oseltamivir carboxylate (k_m_) compared with its elimination rate (i.e. flip‐flop kinetics) 7. Different absorption models, including zero‐order, first‐order, parallel first‐order, sequential zero‐order and first‐order absorption, with and without lag time, were assessed. A transit compartment absorption model was also evaluated, whereby a mean transit time (MTT) represents an average drug transit time through a number (*n*) of hypothetical transit compartments 24.

A linear relationship between creatinine clearance (CL_CR_) and CL/F_OC_ was incorporated *a priori* into the base model 3, 25. All other covariates, including total body weight (TBW), body surface area (BSA) 26, predicted normal weight (PNW) 27, ideal body weight (IBW) 28, semi‐mechanistically derived fat‐free mass (FFM) 29, fat mass, age, dosing (75 mg and 150 mg oseltamivir), gender and obesity, were selected based on physiological plausibility and statistical significance (i.e. *P* < 0.05 from Pearson's, Spearman's and/or Kendall's parameter–covariate correlation tests) and evaluated using a stepwise addition (*P* < 0.05) and backward elimination (*P* < 0.001) approach. Continuous covariates, centred on the median value of the population, were evaluated as linear, exponential and power relationships. Categorical covariates were evaluated as a proportional difference between groups. CL_CR_ (i.e. Cockcroft–Gault) and body size descriptors (BMI, BSA, PNW, FFM and fat mass) were calculated using the following equations:

(2) $${\mathrm{CL}}_{\mathrm{CR}}=\frac{\left(140–\mathrm{age}\right)\times \left[\mathrm{TBW}\;\text{or}\;\mathrm{FFM}\right]\times \left[0.85\;\text{if}\;\text{female}\right]}{72\times \text{serum}\;\text{creatinine}\;\mathrm{in}\;\mathrm{mg}{\mathrm{dl}}^{-1}}$$

(3) $$\mathrm{BMI}=\mathrm{TBW}/{\left(\text{height}\;\mathrm{in}\;m\right)}^{2}$$

(4) $$\mathrm{BSA}=\sqrt{\left[\left(\text{height}\;\mathrm{in}\;\mathrm{cm}\times \mathrm{TBW}\right)/3600\right]}$$

(5) $$\mathrm{PNW}\;\left(\text{male}\right)=1.57\times \mathrm{TBW}–0.0183\times \mathrm{BMI}\times \mathrm{TBW}–10.5$$

(6) $$\mathrm{PNW}\left(\text{female}\right)=1.75\times \mathrm{TBW}–0.0242\times \mathrm{BMI}\times \mathrm{TBW}–12.6$$

(7) $$\mathrm{IBW}=45.4+0.89\times \left(\text{height}\;\mathrm{in}\;\mathrm{cm}-152.4\right)+4.5\;\left(\text{if}\;\text{male}\right)$$

(8) $$\mathrm{FFM}\;\left(\text{male}\right)=\frac{9.27\times 10^{3}\times \mathrm{TBW}}{6.68\times 10^{3}+216\times \mathrm{BMI}}$$

(9) $$\mathrm{FFM}\;\left(\text{female}\right)=\frac{9.27\times 10^{3}\times \mathrm{TBW}}{8.78\times 10^{3}+244\times \mathrm{BMI}}$$

(10) Fatmass=TBW–FFM

A full covariate approach was also employed to investigate whether obesity expressed by different body size descriptors (obesity as a categorical covariate and TBW, BMI, BSA, PNW, IBW, FFM and fat mass as continuous covariates) had an effect on pharmacokinetic parameters. In this approach, the covariate of interest was simultaneously included on all parameters except on relative oral bioavailability, and the model was bootstrapped (*n* = 200) to evaluate the impact of the covariate.

Model selection was guided by evaluation of the OFV and the goodness of fit (GOF). The final model was assessed using a nonparametric bootstrap methodology (*n* = 1000) and simulation‐based diagnostics (i.e. numerical and visual predictive checks, *n* = 2000).

## Results

Twelve non‐obese (median BMI 22.2 kg m^−2^, range 18.8–24.2 kg m^−2^) and 12 obese (median BMI 33.8 kg m^−2^, range 30.8–43.2 kg m^−2^) volunteers were enrolled and completed the study. Full demographic data are presented elsewhere 18. There were no severe adverse events reported during the study.

A total of 624 venous plasma samples were analysed for oseltamivir and oseltamivir carboxylate levels, resulting in 103 (16.5%) concentrations of oseltamivir and 15 (2.40%) concentrations of oseltamivir carboxylate below the LLOQ.

The disposition pharmacokinetics of oseltamivir and oseltamivir carboxylate were both best described by a one‐compartment model with first‐order absorption and elimination. A two‐compartment disposition model of oseltamivir significantly improved the model fit (∆OFV = −63.7). This model performed better because it improved the fit of the terminal elimination phase when there were data below LLOQ but it resulted in an improbable estimate of terminal half‐life (median 1.24 h for a one‐compartment model and 9.18 h for a two‐compartment model) in contrast to the findings of previous studies 9, 18, 30. Furthermore, a one‐compartment disposition model showed no indication of model misspecification when comparing the observed and simulated fraction of oseltamivir concentrations below the LLOQ. Employing the M3 method resulted in a model fit that was comparable with a model with omitted LLOQ data and was therefore not carried forward. A two‐compartment disposition model of oseltamivir carboxylate did not significantly improve the model fit.

**Figure 1:**
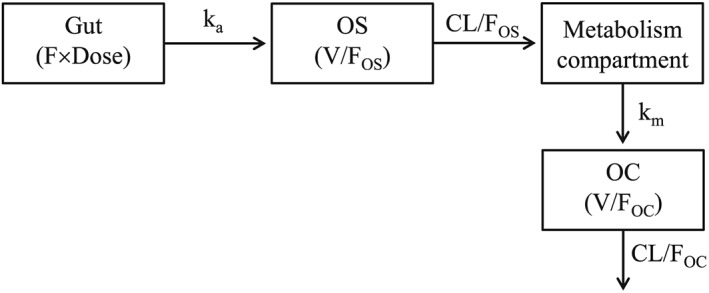
Structural model representation of the final population pharmacokinetic model for oseltamivir and oseltamivir carboxylate in non‐obese (*n* = 12) and obese (*n* = 12) Thai volunteers. Oseltamivir was absorbed from the gut compartment with first‐order absorption to the central compartment. It was metabolized into oseltamivir carboxylate via a metabolism compartment, and then eliminated from the body. CL/F_OS_, oseltamivir clearance; CL/F_OC_, oseltamivir carboxylate clearance; F, relative oral bioavailability; k_a_, absorption rate constant; k_m_, metabolism rate constant; OC, oseltamivir carboxylate; OS, oseltamivir; V/F_OS_, apparent volume of distribution of oseltamivir; V/F_OC_, apparent volume of distribution of oseltamivir carboxylate

A transit‐compartment (*n* = 9) absorption model was significantly better than a first‐order absorption model (∆OFV > −16.2) but it had a substantially increased computational time (>20‐fold increase) and no improvement in goodness‐of‐fit diagnostics. A first‐order absorption model described the absorption data adequately and was therefore carried forward. A metabolism compartment added to accommodate a slower formation rate of oseltamivir carboxylate compared with its elimination rate resulted in a significantly improved model fit (∆OFV = −259). A schematic of the final structural model is provided in Figure 1. Relative bioavailability (F), fixed to unity for the population, was evaluated to allow interindividual variability in the absorption of oseltamivir. Refinement of the variance models resulted in interindividual variability only on F, CL/F_OS_ and V/F_OS_. All other interindividual variability components were estimated to be low (<10%) and did not result in a worse model fit when removed. Implementation of interoccasion variability on k_a_, k_m_ and V/F_OS_ improved the model significantly.

There was no evidence of dose dependency for oseltamivir or oseltamivir carboxylate. A covariate model with body weight implemented as an allometric function on volume and clearance parameters did not improve the model fit compared with the base model. Implementing CL_CR_, calculated using FFM (CL_CR(FFM)_), as a linear function on CL/F_OC_ resulted in a better model fit (∆OFV = −5.84) compared with using TBW (CL_CR(TBW)_, ∆OFV = −1.43). This covariate relationship was carried forward owing to its strong biological prior. Fat mass on CL/F_OS_ (exponential relationship) and PNW on V/F_OS_ (exponential relationship) were significant covariates in the forward step (*P* < 0.05) and could be retained in the backward elimination step at *P* < 0.01 but were both removed using a stricter *P*‐value cut‐off of 0.001 owing to the small sample size. The following equation describes the final covariate model of CL/F_OC_:

(11) $$\mathrm{CL}/F_{\mathrm{OC}\;}=20.6\times \left[1+0.384\times \left({\mathrm{CL}}_{\mathrm{CR}\left(\mathrm{FFM}\right)}-73\right)\right]$$

The estimated final median population parameters with 95% confidence intervals (CIs) obtained from the bootstrap analysis are shown in Table 1. The parameter estimates obtained from the final model were close to the estimates obtained from the bootstrap analysis. Secondary parameters [i.e. maximum concentration (C_max_), time to maximum concentration (T_max_), terminal elimination half‐life (T_1/2)_, total area under the plasma concentration–time curve from time zero to 24 h (AUC_0–24_)] calculated from the individual empirical Bayes *post hoc* estimates of the primary pharmacokinetic parameters in non‐obese and obese individuals are shown in Table 2.

**Table 1:** Population pharmacokinetic parameter estimates from the final model in obese and non‐obese Thai volunteers

**Parameters**	**Population estimate** * **(%RSE** † **)**	**95% CI** †	**IIV/IOV (%CV)** * **(%RSE** † **)**	**95% CI** †
**Oseltamivir**				
**F (%)**	100 (fixed)	‐	17.6 (35.1)	10.6–23.2
**k**_**a**_ **(** **h** ^**–**^^**1**^ **)**	2.81 (10.3)	2.26–3.40	98.7 (19.7)‡	74.4–128
**CL/F**_**OS**_ **(l h** ^**−1**^ **)**	585 (4.91)	532–643	16.6 (48.8)	7.23–23.8
**V/F**_**OS**_ **(l)**	1110 (5.87)	995–1250	18.6 (48.2)‡	10.1–27.3
**k**_**m**_ **(** **h** ^**–**^^**1**^ **)**	2.13 (6.93)	1.90–2.48	43.2 (23.8)‡	31.2–53.2
**Additive residual error**	0.431 (4.01)	0.397–0.465	‐	‐
**Oseltamivir carboxylate**				
**CL/F**_**OC**_ **(l h** ^**−1**^ **)**	20.6 (3.79)	19.2–22.3	‐	‐
**V/F**_**OC**_ **(l)**	159 (4.93)	146–176	18.7 (28.7)	12.3–23.5
**Additive residual error**	0.161 (6.41)	0.140–0.180	‐	‐
**Covariate effects**				
**Effect of CL**_**CR**_ **on CL/F**_**OC**_ **(% change per 10 units of CL**_**CR**_ **)**	3.84 (47.0)	0.178–8.02	‐	‐

CL_CR_, creatinine clearance; CL/F_OS_ , oseltamivir clearance; CL/F_OC_, oseltamivir carboxylate clearance; F, relative bioavailability; k_a_, absorption rate constant; k_m_, metabolism rate constant; V/F_OS_, apparent volume of distribution of oseltamivir; V/F_OC_, apparent volume of distribution of oseltamivir carboxylate.

* Population mean parameter estimates computed from NONMEM. Interindividual variability (IIV) and interoccasion variability (IOV) were calculated as [exp(estimate)–1]^1/2^ × 100.

† Relative standard errors (RSE) and 95% confidence intervals (CIs) were computed from the nonparametric bootstrap method of the final pharmacokinetic model (*n* = 1000).

‡ Interoccasion variability (IOV).

**Table 2:** Secondary parameters of oseltamivir and oseltamivir carboxylate from the final population pharmacokinetic model in obese and non‐obese Thai volunteers

**Secondary parameters [median (range)]**	**Non‐obese (*n* = 12)**		**Obese (*n* = 12)**	
	**75 mg dose**	**150 mg dose**	**75 mg dose**	**150 mg dose**
**Oseltamivir**				
**C**_**max OS**_ **(ng ml** ^**−1**^ **)**	45.1 (31.7–56.7)	103 (57.9–154)	41.8 (21.9–62.3)	90.2 (38.7–139)
**T**_**max OS**_ **(h)**	0.819 (0.444–1.26)	0.630 (0.270–1.62)	0.681 (0.374–1.64)	0.695 (0.410–2.15)
**T**_**1/2 OS**_ **(h)**	1.42 (1.07–1.93)	1.27 (1.01–1.68)	1.37 (0.900–2.32)	1.17 (0.816–1.77)
**AUC**_**0‐24 OS**_ **(h × ng ml** ^**−1**^ **)**	142 (92.9–174)	285 (186–349)	122 (77.6–169)	245 (155–337)
**Oseltamivir carboxylate**				
**C**_**max OC**_ **(ng ml** ^**−1**^ **)**	266 (169–340)	558 (355–734)	265 (169–404)	567 (347–840)
**T**_**max OC**_ **(h)**	4.88 (4.25–6.26)	4.47 (3.42–5.60)	4.58 (3.42–6.42)	4.29 (3.48–6.70)
**T**_**1/2 OC**_ **(h)**	5.45 (4.26–7.91)	5.45 (4.26–7.91)	5.12 (3.72–6.36)	5.12 (3.72–6.36)
**AUC**_**0–24 OC**_ **(h × ng ml** ^**−1**^ **)**	3160 (2380–3800)	6320 (4810–7660)	2870 (2110–4770)	5990 (4300–9720)

Secondary parameter estimates were calculated from the individual empirical Bayes *post hoc* estimates of the primary pharmacokinetic parameters. AUC_0–24_, total area under the plasma concentration–time curve from time zero to 24 h; C_max_, maximum concentration; OC, oseltamivir carboxylate; OS, oseltamivir; T_max_, time to maximum concentration; T_1/2_, terminal elimination half‐life.

**Figure 2:**
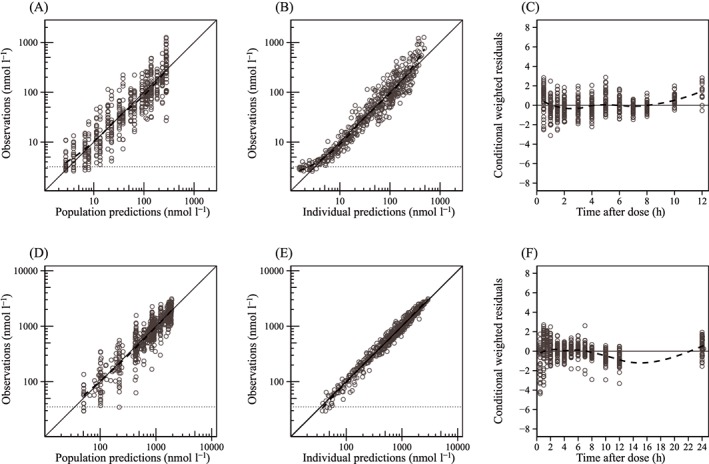
Goodness‐of‐fit diagnostics of the final population pharmacokinetic model of oseltamivir (A, B and C) and oseltamivir carboxylate (D, E and F) in non‐obese (*n* = 12) and obese (*n* = 12) Thai volunteers. Broken lines, locally weighted least‐squares regressions; solid lines, lines of identity; dotted horizontal lines, lower limits of quantification. The observed concentrations, population predictions and individual predictions were transformed into their logarithms (base 10)

**Figure 3:**
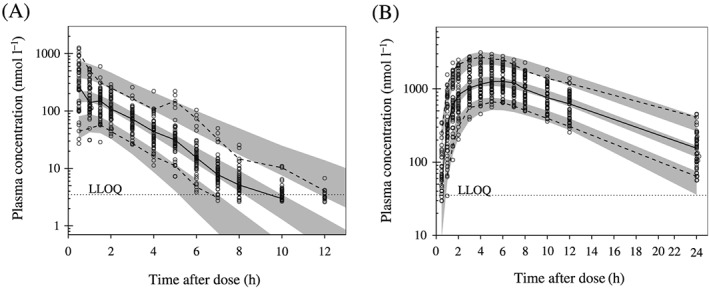
Visual predictive check of the final population pharmacokinetic model of oseltamivir (A) and oseltamivir carboxylate (B) in non‐obese (*n* = 12) and obese (*n* = 12) Thai volunteers. Open circles, observed data; solid lines, 50th percentiles of the observed data; broken lines, 5th and 95th percentiles of the observed data; shaded areas, 95% confidence intervals of simulated (*n* = 2000) 5th, 50th and 95th percentiles; dotted horizontal lines, lower limits of quantification (LLOQ). Concentrations were transformed into their logarithms (base 10)

Epsilon shrinkage was small (6.76%) in the final model. Low eta shrinkages were observed for CL/F_OS_ (9.78%), V/F_OC_ (2.69%) and F (3.46%). Higher eta shrinkages could be seen for V/F_OS_ (29.2% for standard dose and 40.4% for double dose), k_a_ (21.0% for standard dose and 8.88% for double dose), k_m_ (37.9% for standard dose and 19.4% for double dose). Basic goodness‐of‐fit plots for oseltamivir and oseltamivir carboxylate showed an adequate description of the observed data (Figure 2). The visual and numerical predictive check (Figure 3) resulted in 1.92% (95% CI 0.576%, 5.57%) and 3.65% (95% CI 0.768%, 4.80%) of the observed oseltamivir concentrations outside the 5th and the 95th simulated percentiles, respectively, and 2.46% (95% CI 0.493%, 5.58%) and 1.48% (95% CI 0.493%, 5.58%) of the observed oseltamivir carboxylate concentrations outside the 5th and the 95th simulated percentiles, respectively. This suggested that the developed model had adequate predictive power.

**Figure 4:**
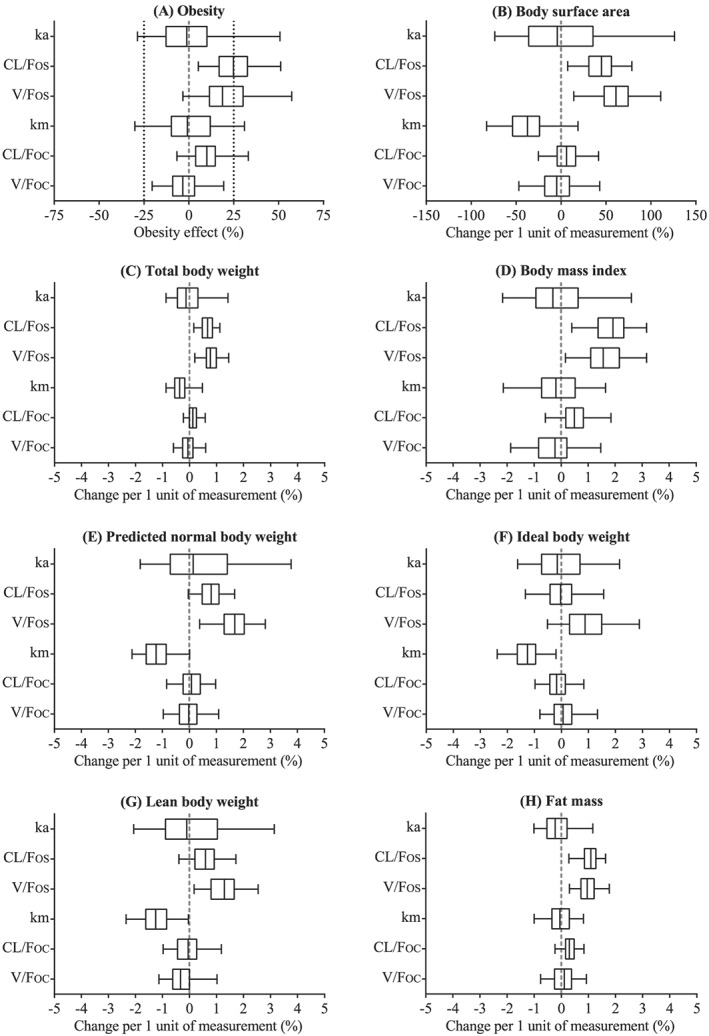
Box plots (interquartile ranges with 2.5 to 97.5 percentiles) displaying the effect of obesity and body size measurements on pharmacokinetic parameters in the full covariate approach. CL/F_OS_, oseltamivir clearance; CL/F_OC_, oseltamivir carboxylate clearance; F, relative oral bioavailability; k_a_, absorption rate constant; k_m_, metabolism rate constant; V/F_OS_, apparent volume of distribution of oseltamivir; V/F_OC_, apparent volume of distribution of oseltamivir carboxylate

The full covariate model analysis demonstrated that obese individuals had an approximately 25% (95% CI 24%, 28%) higher CL/F_OS_, a 20% (95% CI 19%, 23%) higher V/F_OS_ and a 10% (95% CI 9%, 11%) higher CL/F_OC_ compared with non‐obese individuals (Figure 4A). Similarly, CL/F_OS_ and V/F_OS_ increased with increasing BSA, TBW, BMI, PNW, FFM and fat mass (Figure 4). The full covariate approach for BMI and fat mass also suggested a small (<1%) increase in CL/F_OC_ per unit increase in body size.

## Discussion

The prevalence of obesity has increased dramatically over the past two decades and is expected to continue to increase in the future. Based on WHO global estimates, over 200 million men and nearly 300 million women aged ≥20 were obese in 2008 31. The observed increased morbidity and mortality in obese patients infected with A(H1N1)pdm09 influenza virus may result from underlying cardiovascular or respiratory diseases, increased viral expansion, a reduced immune response or altered pharmacokinetics. Oseltamivir is the primary treatment of choice for most types of influenza, including the highly pathogenic H5N1 and the new emerging H7N9 avian influenza.

The present study is the first population pharmacokinetic analysis of oseltamivir and oseltamivir carboxylate to include both non‐obese and obese individuals. A one‐compartment disposition model was found the best to describe the plasma concentration–time data for both oseltamivir and oseltamivir carboxylate. Two‐compartment models have been used previously to describe oseltamivir disposition as they give better goodness of fit in the terminal elimination phase when there are data below the LLOQ 20, 25. However, we found that a two‐compartment model resulted in an unrealistic estimate of terminal half‐life in contrast to the findings of previous studies. Oseltamivir was assumed to be metabolized completely into oseltamivir carboxylate. A metabolism compartment was added to accommodate the formation rate‐limited kinetics of oseltamivir carboxylate which had been successfully implemented in previous studies 32, 33, 34. Secondary pharmacokinetic parameters from the final model were comparable with the noncompartmental analysis results 18 and with those in the literature 20, 30.

Renal excretion is the primary elimination pathway for oseltamivir carboxylate and dose adjustment is recommended in patients with renal impairment. As anticipated, CL_CR_, calculated using a semi‐mechanistically derived FFM, was a significant predictor of CL/F_OC_. High variability of the covariate effect was noted but we chose to retain the covariate owing to its strong clinical relevance. This finding is in agreement with previously published studies showing that renal function is closely related to lean body mass 35. Using actual body weight for calculating Cockcroft–Gault CL_CR_ therefore overestimates the values in obese patients, which can lead to unnecessary dose adjustments 36, 37. Volunteers in the present study had a calculated CL_CR(FFM)_ between 48.0 ml min^−1^ and 114 ml min^−1^ and an estimated CL/F_OC_ between 17.7 l h^−1^ and 25.8 l h^−1^, resulting in AUC_0–24_ of 4850 h × ng ml^−1^ and 2150 h × ng ml^−1^ (75 mg dose), respectively. The developed model predicted a 9–15% increased exposure to oseltamivir carboxylate for volunteers with a CL_CR(FFM)_ between 30 ml min^−1^ and 50 ml min^−1^, and >24% increased exposure for volunteers having CL_CR(FFM)_ < 10 ml min^−1^, compared with a normal CL_CR(FFM)_ of 75 ml min^−1^. Volunteers with severe renal impairment were not included in the present study, which limits the extrapolation of the results to this population. However, a recently published study has also confirmed the impact of CL_CR_, calculated using a semi‐mechanistically derived FFM, in individuals with renal impairment 34.

Obesity did not have a significant impact on any oseltamivir or oseltamivir carboxylate pharmacokinetic parameters in the formal covariate analysis. However, the full covariate analysis demonstrated a relatively higher apparent CL/F_OS_ and apparent V/F_OS_ in obese individuals and a modest increase in the apparent CL/F_OC_. Higher CL/F_OS_ may suggest increased CES1 activity. Previously published studies have suggested a higher expression of CES1 in abdominal subcutaneous adipose tissue from obese adults 38 and in visceral adipose tissue from obese children 39 compared with those from their lean peers. Oseltamivir is a lipophilic compound and was expected to have an increased volume of distribution in obese individuals because of drug distribution into excess body fat, whereas this was unlikely for the hydrophilic oseltamivir carboxylate. The observed altered pharmacokinetic properties of oseltamivir are likely to be of little clinical relevance as oseltamivir is a prodrug of oseltamivir carboxylate, which is responsible for the antiviral effect. The observed increased morbidity and mortality in obese patients infected with A(H1N1)pdm09 cannot, therefore, be explained by altered pharmacokinetic properties, and the data presented here confirm previous work 18 in finding that a dose adjustment in obese patients is not warranted.

A limitation of the present study was the small sample size. However, the study used intensive sampling and included non‐obese individuals and those from the whole spectrum of obesity (BMI range 18.8–43.2 kg m^−2^).

## Conclusion

The population pharmacokinetic properties of oseltamivir and oseltamivir carboxylate were described successfully by a simultaneous parent‐metabolite model using a nonlinear mixed‐effects approach. CL_CR_ had a significant impact on CL/F_OC_, resulting in a proportional decrease in oseltamivir carboxylate exposure with increasing CL_CR(FFM)_. Oseltamivir carboxylate pharmacokinetics were unaffected by obesity. The data presented here confirmed that oseltamivir dose adjustment in obese patients is not necessary on the basis of its pharmacokinetics.

## Competing Interests

This study was supported by the South East Asia Influenza Clinical Research Network and was part of the Wellcome Trust Mahidol–Oxford Tropical Medicine Research Programme funded by the Wellcome Trust of the UK. The funder had no part in the study design, implementation or analysis of the study or in the decision to publish the results. All authors have completed the Unified Competing Interest form at http://www.icmje.org/coi_disclosure.pdf (available on request from the corresponding author) and declare no financial relationships with any organizations that might have an interest in the submitted work in the previous 3 years; no other relationships or activities that could appear to have influenced the submitted work.

We are grateful to the volunteers participating in this study. We also thank the staff of the Hospital for Tropical Diseases, Faculty of Tropical Medicine, Mahidol University.

## Contributors

PJ, ND, NW and SP contributed to study design and conduct of the clinical study. WH performed the drug analysis. KC and JT contributed to the pharmacometric analysis and the drafting of the manuscript. All authors reviewed the manuscript and approved the final version.
